# Bidirectional Effects of Mao Jian Green Tea and Its Flavonoid Glycosides on Gastrointestinal Motility

**DOI:** 10.3390/foods12040854

**Published:** 2023-02-16

**Authors:** Lei Wu, Xiang Jin, Canwen Zheng, Fengjing Ma, Xue Zhang, Pengfei Gao, Jianhua Gao, Liwei Zhang

**Affiliations:** 1 Institute of Molecular Science, Shanxi University, Taiyuan 030006, China; 2 Shanxi Key Laboratory of Minor Crops Germplasm Innovation and Molecular Breeding, College of Life Sciences, Shanxi Agricultural University, Jinzhong 030801, China; 3 College of Animal Science, Shanxi Agricultural University, Jinzhong 030801, China

**Keywords:** *Dracocephalum rupestre* Hance, gastric emptying, small intestine propulsion, muscle contractility, gastrointestinal hormones, liquid chromatography, gut microbiota

## Abstract

Mao Jian Tea (MJT) has been generally consumed as a digestive aid for more than a hundred years in the Shanxi province of China. However, determination of its efficacy still remains elusive. This study investigated the effect of Mao Jian Green Tea (MJGT) on gastrointestinal motility. The biphasic effects of the hydro extracts of MJGT on gastric emptying and small intestinal propulsion of rats were identified in vivo; namely, the low (MJGT_L) and medium (MJGT_M) concentrations promoted gastrointestinal motility (*p* < 0.05), whereas the high concentration (MJGT_H) showed the opposite effect (*p* < 0.01). The expression levels of the gastric hormones, GAS, MTL and VIP (*p* < 0.05) were consistent with the gastrointestinal motility variation, with the exception of MTL in MJGT_H group (*p* > 0.01). Two flavonoids, eriodictyol (0.152 mg/mL) and luteolin (0.034 mg/mL), and the corresponding glycosides eriodictyol-7-*O*-glucoside (0.637 mg/mL) and luteolin-7-*O*-glucoside (0.216 mg/mL), dominated the hydro extracts identified by HPLC and UPLC-ESI-MS. These compounds can regulate the muscle strip contractions isolated from the gastrointestinal tissues. Additionally, the different concentrations also influenced the gut microbiota accordingly characterized by 16S rDNA gene sequencing. The MJGT_L boosted several probiotic bacteria, such as Muribaculaceae (1.77-fold), Prevotellaceae (1.85-fold) and Lactobacillaceae (2.47-fold), and suppressed the pathogenic species such as Staphylococcaceae (0.03-fold) that, conversely, was enriched in the MJGT_H group (1.92-fold). Therefore, the biphasic effect indicated that the dosage of the herbal tea should not be overlooked.

## 1. Introduction

In modern times, the nutritional and medicinal contents of different herbal teas have been revealed, and their potential for use in preventive health care has been driving a huge market. At present, China is the largest producer and consumer of herbal teas, but the market has also spread into many regions of the world including the Asia-Pacific, North America, Latin America, Europe, the Middle East and Africa, which has led to an upsurge in research on the effects of different herbal teas and their action modes [1,2,3,4]. One example of a traditional Shanxi herbal tea, named Mao Jian tea (MJT), has been prevailing in the Shanxi Province of northern China. In this region, several coarse grains such as oat (*Avena sativa*), buckwheat (*Fagopyrum esculentum*) and common millet (*Panicum miliaceum*) are the routine foods of local residents, but these foods are relatively difficult to digest. Therefore, people drink the MJT to promote the digestion and prevent the retention of foods.

MJT is made from the whole grass of a perennial herb, *Dracocephalum rupestre* Hance (also known as Yan Qing Lan or Mao Jian Cao in Chinese), of the family Lamiaceae, and is different with the famous Xinyang Maojian tea of Henan province in China [5]. Mao Jian Cao (MJC) is mainly distributed in Shanxi and Qinghai provinces and Inner Mongolia Autonomous Region in China, and was first recorded as medicine in the “Highland herbal medicine treatment manual” [6]. Several therapy functions of MJC were discovered, including relieving headaches, soothing sore throats, subsiding coughs and preventing icterohepatitis [7]. Study has also indicated that MJC is rich in flavonoids and terpenoids [8]. At present, MJT is divided into black tea (MJBT) and green tea (MJGT) variants. The former requires a longer fermentation time and is a traditional product of the local population with a slightly sweet and mellow taste. The MJGT is a new product that mimics the process of making green tea, which helps retain more of its original nutritional and medicinal components, especially the multifunctional polyphenolic compounds [9,10,11]. However, its activity and corresponding modes of action on aiding the digestive system have not yet been investigated.

In this study, we focused on the mechanism of action of MJT on the gastrointestinal tract. The effects of the hydro extracts of MJGT with different concentrations on improving small intestine propulsion and gastric emptying were determined in rats. The responses of the isolated muscle strips to the MJGT were further identified by monitoring the fluctuations of their contractions. Finally, the variations of the intestinal microbial diversity were analyzed by 16S rDNA gene sequencing. We found that the medium and lower dosage promoted gastrointestinal motility, whereas the high dosage caused movement inhibition.

## 2. Materials and Methods

### 2.1. Sample Collection

MJGT was purchased from Jiufeng Cooperative (Ningwu, Shanxi, China).

### 2.2. Animals

The specific-pathogen-free (SPF)-grade Sprague Dawley (SD) rats (half male and half female, Spelford Biotechnology Co., Ltd., Beijing, China), measuring 200 ± 20 g in weight, were used in this study. Each rat was raised separately in an access-restricted room with controlled temperature (22~24 °C) [12] and even light-dark cycles (12:12 h). Clean water and foods were fed daily to the rats. All animal work was approved by the Experimental Animal Ethical Committees of Shanxi Agricultural University and performed under the regulation and guidelines of the Experimental Animal Ethical Committees of Shanxi Agricultural University (Taigu, China). The permit number of the Ethics code was SXAU-EAW-2018R0319.

### 2.3. The MJGT Production Process

The fresh MJC leaves were incubated at 270 °C for 3 min for fixation, followed with rolling by roller for 45 min, and finally dried at 110 °C for 1.5 h [13].

### 2.4. Animal Grouping and Affects In Vivo Gastrointestinal Motility

Before administration, the rats were acclimated for a week and then randomly divided into 5 groups (*n* = 10). The low concentrations (MJGT_L, 17 mg/mL), medium concentrations (MJGT_M, 34 mg/mL), and high concentrations (MJGT_H, 68 mg/mL) of MJGT were oral administered with the hydro extracts for 30 days, and the gavage volume was 1 mL/100 g body weight, respectively. This is equivalent to a 60 kg adult consuming about 2.83 mg/mL of tea a day for MJGT_L. The Mosapride citrate hydrate (MSP, 1 mg/kg) was used for the positive control group, while in the negative control group (NC), the saline with equal volume was used [14]. All of the extracts were stored at 4 °C.

On the 30th day of administration, 300 mL of semi-solid paste was prepared according to the method described previously, with some modification [15]. Briefly, 10 g of sodium carboxymethylcellulose was dissolved in 250 mL of distilled water, and afterward, 16 g of milk powder, 8 g of glucose, 8 g of starch and 2 g of activated charcoal powder was added into this solution. This was mixed thoroughly to obtain the semi-solid paste. Forty min after the last drug administration, each rat was intragastrically administrated with 2 mL of freshly prepared semi-solid paste that was weighed in advance (Mp). Twenty minutes later, the stomachs and small intestines of these rats were rapidly removed. The full stomach weight (Mfs), the net stomach weight (Mns), the total length of the small intestine (Lt), and the carbon propulsive distance (Lc) in the small intestine were measured, individually. The following equations were used for calculating the gastric emptying rate [Equation (1)] and small intestine propulsion rate [Equation (2)].

Gastric emptying rate (%) = [1 – (Mfs – Mns)/Mp] × 100%(1)

Small intestine propulsion rate (%) = (Lc/Lt) × 100%(2)

### 2.5. Sampling of Serums and Colonic Contents

The other batch of rats with the same grouping and administration protocols were used to determine the serums and colonic contents. On the day that dosing ended, 10 mL of the cardiac puncture blood of each rat was collected [16] and centrifuged immediately at 2000 rpm at 4 °C for 15 min. Moreover, the feces were taken from the colon of each rat and frozen rapidly in liquid nitrogen. The obtained samples were stored in autoclaved freezing tubes at −80 °C.

### 2.6. Muscle Contractility In Vitro

The non-administrated rats were fasting without water for 24 h before the experiment, and then anesthetized with pentobarbital sodium (40 mg/kg). The abdomen of each rat was opened along the midline and the stomach, fundus, duodenum, jejunum and ileum were removed for muscle strip separation. Approximately 2 cm of each muscle strip was soaked in 20 mL pre-oxygenated (95% O_2_ and 5% CO_2_) Tyrode’s solution (146 mmol/L NaCl, 3 mmol/L KCl, 12 mmol/L NaHCO_3_, 1 mmol/L MgCl_2_, 0.5 mmol/L NaH_2_PO_4_, 1.5 mmol/L CaCl_2_, and 5 mmol/L D-glucose, pH = 7.35 ± 0.05) at 37 ± 0.5 °C [17]. After being fixed in a multichannel physiological recorder (Chengdu Instrument Factory, China), the contractile responses of isolated muscle strips from the four organs against the hydro extracts of MJGT were recorded simultaneously at 1.5 g initial tension. Before measurement, all tissues were equilibrated for 1 h. Each isolated tissue was exposed in an orderly way to the 20 mL Tyrode’s solution containing the extra 80 μL of MJGT_L (L), 160 μL of MJGT_L (M) and 320 μL of MJGT_L (H) or five concentrations (S1 to S5, 0.797 μg/mL to 12.030 μg/mL) of each flavonoid standard added with similarly cumulative manner (Table S1) [18].

### 2.7. The Expression Level of Gastrointestinal Hormones in Serum

The expression levels of motilin (MTL), gastrin (GAS) and vasoactive intestinal peptide (VIP) were determined by double-antibody sandwich enzyme-linked immunosorbent assay (ELISA) using Rat MTL (Motilin) ELISA Kit (D731179), Rat GT (Gastrin) ELISA Kit (D731177) and Rat VIP ELISA Kit (D731185) of Sangon Biotech Co., Ltd. (Shanghai, China), respectively, according to the corresponding manufacturers’ instructions.

### 2.8. Identification of Active Ingredients

The dried MJGT was crushed into powder and passed through a sieve with 40-mesh (0.425 mm in aperture size).

Preparation of test solution of hydro extract: An amount of 10 mL of double deionized water (ddH_2_O) was added into 0.1700 g of sieved MJGT powder and weighed. The mixture was boiled for 20 min and the lost weight was made up with fresh water. Afterwards, the insoluble particles in the mixture were eliminated by passing through the filter paper and 0.22 μm filtration membrane in turn.

Preparation of methanol soluble sample in hydro extract: An amount of 8.5365 g of sieved MJGT powder was weighed in a 1000 mL distillation flask, then 500 mL of ddH_2_O was added, and the mixture incubated for 20 min after boiling. The mixture was filtrated using the method mentioned above, the filtrate was dried using a rotary evaporator, and the extract was divided into three parts and weighed, respectively. For each part, the resuspended solution with 1 mg/mL concentration was prepared using 50% methanol and filtered with a 0.22 μm filter membrane.

Preparation of standard solutions: Standard stock solutions of four flavonoids including eriodictyol-7-O-glucoside (0.40 mg/mL), eriodictyol (0.25 mg/mL), luteolin-7-O-glucoside (0.22 mg/mL) and luteolin (0.196 mg/mL) were prepared using methanol as solvent. Six dilutions (2-, 4-, 5-, 10-, 20- and 100-fold) of each standard stock solution with methanol were used to determine the standard curve. For each dilution, 1 mL solution was injected into the column system.

HPLC conditions: The test solution and the standard solutions were loaded on an Agilent 1200 liquid chromatography system, equipped with a quaternary solvent delivery system, an autosampler and a DAD detector, for determining the corresponding compounds and their contents. The separation was carried out on an Agilent TC-C18 column (250 mm × 4.6 mm, 5 μm), according to the method established in our laboratory. In brief, the analysis was carried out by gradient elution with a mobile phase that consisted of solvent A (0.3% aqueous acetic acid, *v/v*, HPLC grade, Tianjin Comio Chemical Reagent Co., Ltd., Tianjin, China) and solvent B (methanol, HPLC grade, Thermo Fisher Scientific Co., Ltd., Beijing, China). Elution gradients were: 0~22 min, 32% B; 22~23 min, 32~37% B; 23~36 min, 37% B; 36~37 min, 37~45% B; 37~46 min, 45% B; 46~47 min, 45~60% B; 47~60 min, 60~80% B. UV absorption of eriodictyol and eriodicty-7-O-glucoside was monitored at 284 nm, while monitoring of luteolin and luteolin-7-O-glucoside was at 350 nm. The column temperature was set at 25 °C. The flow rate was 1.0 mL/min and the sample injection volume was 10 μL.

UPLC-ESI-MS conditions: The mobile phase consisted of solvent A (0.1% formic acid, *v/v*, MS grade, Thermo Fisher Scientific Co., Ltd., Beijing, China) and solvent B (methanol, MS grade, Thermo Fisher Scientific Co., Ltd., Beijing, China). Elution gradients were: 0 min, 10% B; 0~18 min, 10~35% B; 18~26 min, 35~45% B; 26~27 min, 45~10% B; 27~30 min, 10% B. The flow rate was 0.2 mL/min. The injection volume was 5 μL. The column temperature was 40 °C. An Acquity UPLC^®^ BEH C18 column (2.1 mm × 100 mm, 1.7 μm, Waters, Ireland) was used. Sample analysis was performed on a ThermoDionex UltiMate 3000 high-performance liquid chromatograph coupled to a Thermo Q Exactive HF hybrid quadrupole-Orbitrap mass spectrometer (Thermo Fisher Scientific, St. Louis, MO, USA), and operating in both positive and negative ion modes. The capillary temperature, sheath gas flow and auxiliary gas flow were set at 330 °C, 35 (arbitrary unit, arb) and 12 (arb), respectively. Survey full-scan MS spectra (mass range m/z 150 to 1500) were acquired with resolution R = 70,000 and AGC target 1 × 10^6^. The stepped normalized collision energies (NCEs) were set to 20, 40 and 60, respectively.

### 2.9. 16S rDNA Gene Sequencing of Fecal Samples

DNA extraction and PCR amplification: The feces were taken from the colon of each rat and frozen rapidly in liquid nitrogen. Microbial community genomic DNA of the rat fecal samples was extracted using the E.Z.N.A.^®^ soil DNA Kit (Omega Bio-tek, Norcross, GA, USA) according to manufacturer’s instructions. The qualities of DNA extracts were examined by the agarose gel electrophoresis and NanoDrop 2000 UV-VIS spectrophotometer (Thermo Scientific, Wilmington, DE, USA). DNA samples that passed the quality test were used as templates for PCR detection. The target site of hypervariable region V3-V4 of the bacterial 16S rDNA gene were amplified using primer pairs 338F (5′-ACTCCTANCGGAGGCAGCAG) and 806R (5′-GGACTACHVGGGTWTCTAAT) with the reaction condition as follows: initial denaturation at 95 °C for 3 min, followed by 27 cycles of denaturing at 95 °C for 30 s, annealing at 55 °C for 30 s and extension at 72 °C for 45 s, and single extension at 72 °C for 10 min, and preserved at 10 °C. The mixture for each reaction contained 5 × TransStart FastPfu buffer 4 μL, 2.5 mM dNTPs 2 μL, each primer (5 μmol/L) 0.8 μL, TransStart FastPfu DNA Polymerase 0.4 μL, and template DNA 10 ng. The double deionized water was used to make up the final volume to 20 μL. The PCR reactions were performed in triplicate. Each PCR product was separated by 2% agarose gel, and then extracted and purified using the AxyPrep DNA Gel Extraction Kit (Axygen Biosciences, Union City, CA, USA) according to manufacturer’s instructions and quantified using Quantus Fluorometer (Promega, Madison, WI, USA).

Illumina MiSeq sequencing: Purified amplicons were pooled in equimolar and paired-end sequenced on an Illumina MiSeq PE300 platform/NovaSeq PE250 platform (Illumina, San Diego, CA, USA) according to the standard protocols by Majorbio Bio-Pharm Technology Co. Ltd. (Shanghai, China). The raw reads were deposited into the NCBI Sequence Read Archive database (Accession Number: PRJNA755509).

Processing of sequencing data: The raw 16S rDNA gene sequencing reads were demultiplexed, quality-filtered by fastp (v0.20.0) [18] and merged by FLASH (v1.2.7) [19]. Three criteria were followed for data processing. The first of these was sequence filtering. The reads were truncated at any site receiving a low average quality score (<20) over a 50 bp sliding window. The truncated reads shorter than 50 bp and reads containing ambiguous characters were discarded. Second was assembly. Only paired sequences with overlapping longer than 10 bp were assembled. The maximum mismatch ratio of overlap region was 0.2. Thirdly, samples and the sequence direction were distinguished according to the barcodes and primers. The exact barcode matching and low primer mismatch (<3) in matching were required.

Operational taxonomic units (OTUs) with 97% similarity cutoff were clustered using UPARSE (v7.1) [20], and chimeric sequences were identified and removed. The taxonomy of each OTU representative sequence was analyzed by RDP Classifier (v2.2) [21] against the 16S rDNA database, using a confidence threshold of 0.7.

### 2.10. Data Analysis

One-way ANOVA and Duncan’s multiple range test were performed for post-hoc evaluations by SPSS25 (IBM, Armonk, New York, NY, USA). The differences were statistically significant at *p* < 0.05 or 0.01. Data were expressed as Mean ± SEM.

## 3. Results

### 3.1. MJGT Hydro Extracts Regulated the Gastric Emptying and Small Intestinal Propulsion In Vivo

**Figure 1:**
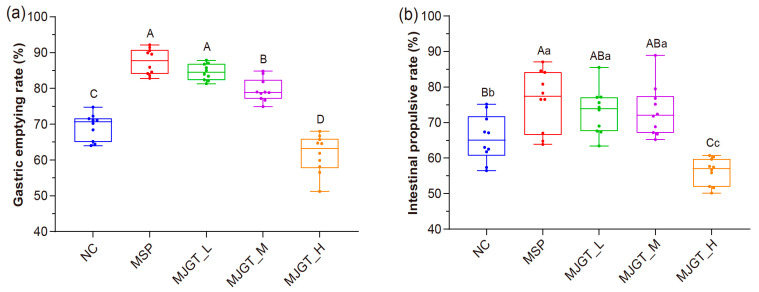
Effect of different concentrations of MJGT hydro extracts on gastric emptying rate (**a**) and intestinal propulsive rate (**b**) in vivo. *n* = 10 rats for each group. Capital and lowercase letters above the bars indicate the difference significance at 0.01 or 0.05 level, respectively.

The efficiency of gastric emptying and small intestinal propulsion directly reflects the rate of food propulsion in the digestive system. Therefore, we first determined the effect of MJGT hydro extracts on both movements in vivo in rats. Compared with the NC group, MJGT_L and MJGT_M not only significantly reduced food retention in the stomach (*p* < 0.01, Figure 1a), but also promoted food propulsion in the small intestine (*p* < 0.05, Figure 1b), which were similar results to the positive control MSP, a 5-hydroxytryptamine receptor (5-HT_4_) agonist that can enhance the gastric emptying [14]. Surprisingly, both movements were significantly inhibited by MJGT_H (*p* < 0.01). The results suggested that the regulatory effect of MJGT was able to regulate the gastrointestinal motility, with the dosage related.

### 3.2. Low to Medium Concentration of MJGT Hydro Extracts Promoted Gastrointestinal Hormone Secretion

**Figure 2:**
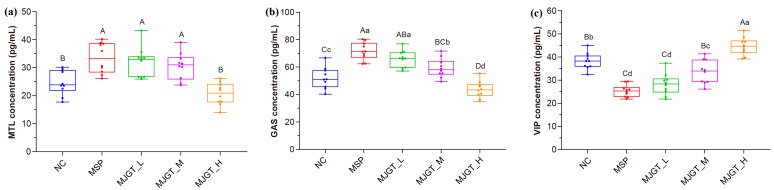
Effects of different concentrations of MJGT hydro extracts on the secretion of MTL (**a**), GAS (**b**) and VIP (**c**) in rat serum after 30 d administration. *n* = 10 for each group. Capital and lowercase letters above the bar indicate the difference significance at 0.01 or 0.05 level, respectively.

Hormones, such as MTL, GAS and VIP, prominently regulate gastrointestinal motility, absorption and digestion. In this study, the concentrations of the positive regulators MTL and GAS were elevated by MJGT_L or MJGT_M treatment (Figure 2a,b) while the accumulation of the negative regulator VIP was downregulated (*p* < 0.05, Figure 2c), consistent with the observation in the MSP group. Notably, the impacts of MJGT_M on GAS and VIP were weaker than that of MJGT_L. As the concentration increased to the high level (MJGT_H), the opposite results were obtained, including the decreased concentrations for MTL and GAS (*p* < 0.01) and an increase for VIP (*p* < 0.01). The observation indicated that the regulatory effect on gastrointestinal motility of MJGT would result from the accumulation of the hormones, at least partially.

### 3.3. Two Flavonoids and Their Glycosides Were the Main Components in MJGT Hydro Extracts

**Figure 3:**
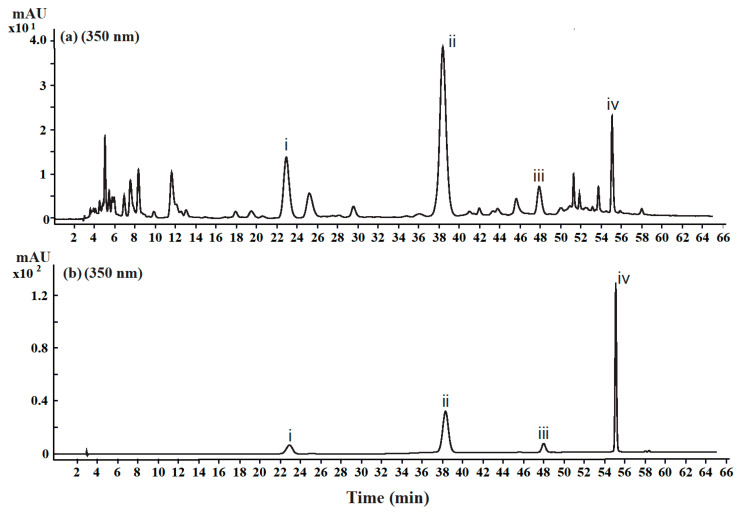
The chromatogram of the MJGT hydro extracts (**a**) and the mixed standards of the four flavonoids (**b**) detected at 350 nm. i: eriodictyol-7-*O*-glucoside; ii: luteolin-7-*O*-glucoside; iii: eriodictyol; iv: luteolin.

Four characteristic absorption peaks were identified as eriodictyol-7-O-glucoside (Figure S1i), luteolin-7-O-glucoside (Figure S1ii), eriodictyol (Figure S1iii) and luteolin (Figure S1iv) by UPLC-ESI-MS (ultra-performance liquid chromatography-electrospray tandem mass spectrometry), based on the mass spectrometry data and fragment-ion information (Figure S2 and Table S2). Afterwards, each compound was verified by comparison with the corresponding standard by high performance liquid chromatography (HPLC) (Figure 3). The contents of four flavonoids (eriodictyol, eriodictyol-7-*O*-glucoside, luteolin and luteolin-7-*O*-glucoside) in MJGT extract were determined by the standard curves of corresponding compounds established by HPLC. The produced curves of four standard compounds showed good linear regression relationships (R^2^ = 0.9991~0.9998) (Table S3), precisions [relative standard deviation (RSD) = 1.09~1.70%], repeatability (RSD = 0.69~1.26%), stability (RSD = 2.13~3.22%), and recovery (94.80~100.58%), indicating that the HPLC method developed in this study was suitable for determining the content of the four flavonoids in the MJGT extract. As a result, the contents of eriodictyol, eriodictyol-7-*O*-glucoside, luteolin and luteolin-7-*O*-glucoside in MJGT_L were 0.152 mg/mL (0.527 mmol/L), 0.637 mg/mL (1.414 mmol/L), 0.034 mg/mL (0.119 mmol/L) and 0.216 mg/mL (0.482 mmol/L), respectively.

To determine the proportions of the four flavonoids in all non-volatile solutes, the MJGT_L was spin-dried, weighed and then re-dissolved in 50% methanol. After filtration by 0.22 μm filter membrane, the solution was analyzed by HPLC. As a result, 2.97 ± 0.01% (eriodictyol), 10.19 ±0.03% (eriodictyol-7-*O*-glucoside), 0.74 ± 0.02% (luteolin) and 3.77 ± 0.03% (luteolin-7-*O*-glucoside) were identified in the dried solutes, respectively (Table 1).

### 3.4. MJGT Hydro Extracts Altered the Muscle Contractility In Vitro

**Figure 4:**
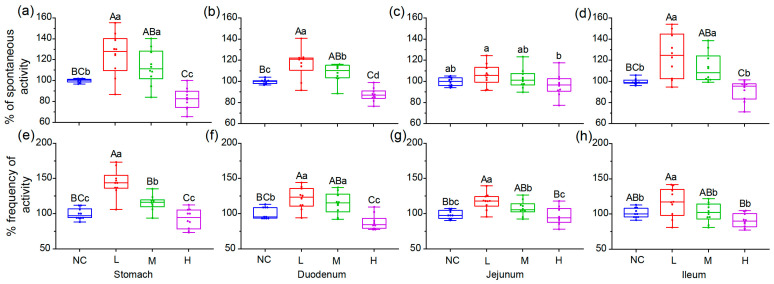
Effects of different concentrations of MJGT hydro extracts on the contraction of isolated muscle strips from different gastrointestinal tissues in vitro. The contraction is indicated by the percentage (%) of spontaneous activity (**a**–**d**) and the corresponding frequency (**e**–**h**) compared to the negative control (NC, 100%) *n* = 10 rats for each group. Capital and lowercase letters above the bar indicate the difference significance at 0.01 or 0.05 level, respectively.

Peristaltic movements of the intestine are essential to propel food through the gastrointestinal tract. These movements are produced by the coordinated contraction and relaxation of the circular and longitudinal smooth muscles that form the outer muscular layer [22]. To determine the action mode of MJGT hydro extracts on the stomach and small intestine, the muscle strips from the fundus of the stomach and various segments of the small intestine were isolated for, respectively, evaluating the muscle contractility indicated by the spontaneous activity (tension), and the corresponding frequency in vitro. The results showed that the low concentrations (L, Table S1) significantly strengthened the spontaneous activity of the muscle strip from each part, with the exception of the strips isolated from jejunum (*p* < 0.01, Figure 4a–d). The treatment with medium concentration (M, Table S1) led to similar results. Interestingly, in the H group, the strong suppression on the corresponding parts of the stomach and duodenum was revealed (*p* < 0.01). The muscle strips of the jejunum and ileum were not sensitive to the highest concentration tested. MJGT hydro extracts also affected the contraction frequency of the isolated muscle tissue (Figure 4e–h). Intensified frequencies were observed for the muscle tissues of the four parts in the L treatment (*p* < 0.05), while the M treatment only affected the counterparts of the stomach and duodenum positively (*p* < 0.01). No significant change in the contraction frequency was obtained during the H treatment, with the exception of the strips isolated from the duodenum (*p* < 0.05). 

### 3.5. The Glycosylation of Flavonoids Promoted the Contractility of Muscle Strips

**Figure 5:**
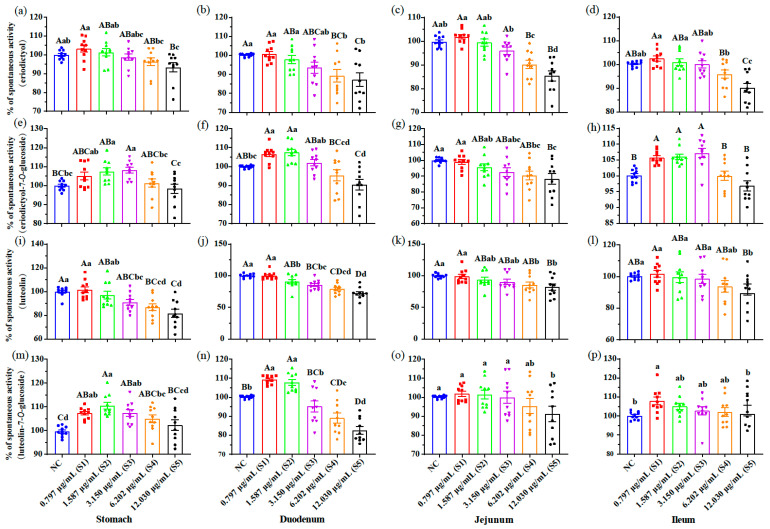
Effects of eriodictyol (**a**–**d**), eriodictyol-7-*O*-glucoside (**e**–**h**), luteolin (**i**–**l**) and luteolin-7-*O*-glucoside (**m**–**p**) at different concentrations on the spontaneous activity of the isolated muscle strips from four gastrointestinal tissues in vitro. The spontaneous activity of the negative control (NC) was considered as 100%. *n* = 10 for each group. Capital and lowercase letters above the bar indicate the difference significance at 0.01 or 0.05 level, respectively.

The impact of the eriodictyol and luteolin, as well as their glycosides, on the isolated muscle strips of the stomachs and intestines of rats were investigated in vitro using the standard compounds. Interestingly, only inhibitory effects on the spontaneous activity of the muscle strips were observed at the high concentrations tested for the aglycones eriodictyol (S4 and S5, *p* < 0.05) and luteolin (from S2 to S5, *p* < 0.05) (Figure 5a–d,i–l). However, the prokinetic effects occurred when treated with the corresponding glucosides at lower concentrations for all tissues except the jejunum (Figure 5e–h,m–p). Notably, the high concentrations (S4 = 6.202 μg/mL or S5 = 12.030 μg/mL) of the eriodictyol-7-*O*-glucoside and luteolin-7-*O*-glucoside also suppressed the spontaneous activity of the muscle strips of the duodenum and jejunum.

No negative impacts were determined, except in the treatment using the highest concentration of eriodictyol-7-*O*-glucoside (S5 = 12.030 μg/mL) for the contraction frequency (Figure S3). Three tissues, including stomach, duodenum and jejunum, were positively stimulated by luteolin-7-*O*-glucoside at low and medium concentrations (S1 = 0.797 μg/mL or S2 = 1.587 μg/mL).

Only the contents of the eriodictyol-7-*O*-glycoside in L (2.538 μg/mL), M (5.056 μg/mL) and H (10.031 μg/mL) treatments were close to the medium and high concentrations (S3 = 3.150 μg/mL, S4 = 6.202 μg/mL and S5 = 12.030 μg/mL) of the corresponding standard compounds, respectively. According to the in vitro results, the prokinetic effect of eriodictyol-7-*O*-glycoside occurred at concentrations less than 3.150 μg/mL (S3), whereas at higher concentrations (S4 and S5), inhibitory effects were observed (Figure 5 and Table S1). The results were consistent with the test treated by MJGT hydro extracts (Figure 4). For eriodictyol and luteolin-7-*O*-glucoside, their contents in L, M and H were close to the S1 (0.797 μg/mL), S2 (1.587 μg/mL) and S3 (3.150 μg/mL), respectively, in which the prokinetic effects were dominant (Figure 5 and Table S1). Additionally, the contents of luteolin in L, M and H were all less than the S1 (0.797 μg/mL) where no significant impact was obtained.

### 3.6. MJGT Hydro Extracts Change the Gut Microbiota of Rat

**Figure 6:**
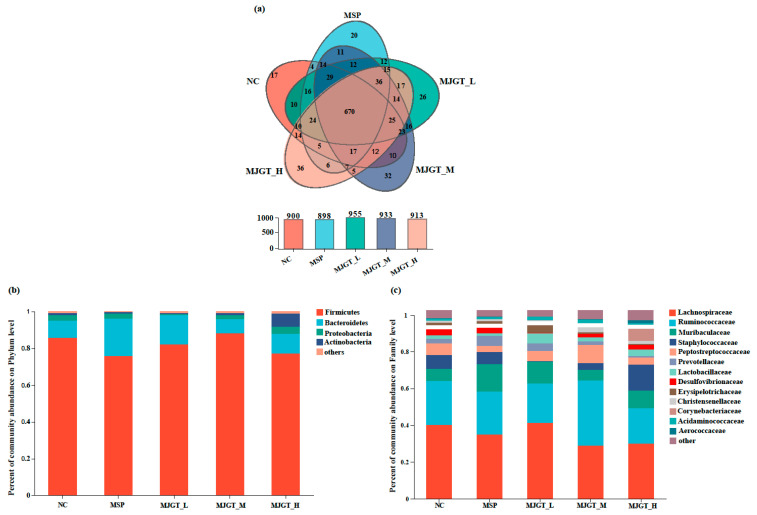
The analysis of the variations of gut microbiota treated by different concentrations MJGT hydro extract. The numbers and the differences of the OTUs among treatments are shown in panel (**a**), and the variations of microbiota composition are also displayed at the phylum (**b**) and family (**c**) level, respectively (*n* = 4 for each group).

To clarify whether the MJGT hydro extracts work on gut microbiota, the 16S rDNAs of rat fecal flora were amplified (Figure S4) and analyzed by high throughput sequencing. The saturated Shannon curve and total number of observed OTUs (Sobs) indicated a sufficiently large coverage for each sample and the data can be used for the downstream analysis (Figure S5). For each group, 898 (MSP) to 955 (MJGT_L) OUTs were determined and a total of 1148 OTUs were obtained from the five treatment groups (Figure 6a). The community diversity decreased as the concentration of MJGT hydro extracts increased. Treatment-specific OTUs were identified in five groups, including NC (17), MSP (20), MJGT_L (26), MJGT_M (32) and MJGT_H (36) (Figure 6a).

**Figure 7:**
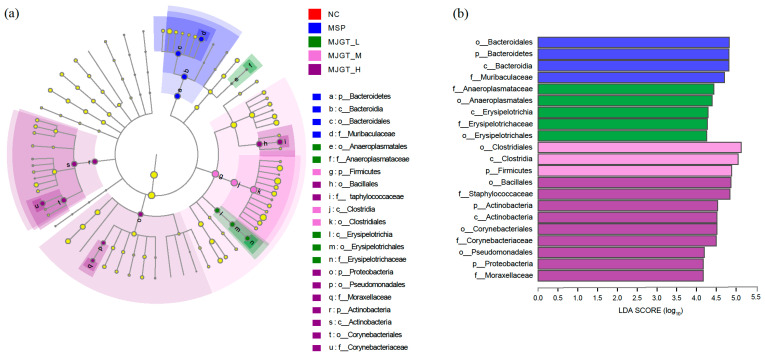
Hierarchical tree map (**a**) and the linear discriminant analysis histogram (**b**) of the gut microbiota in treatments (*n* = 4 for each group).

Linear discriminant analysis Effect Size (LEfSe) revealed significant differences in the dominance of Bacteroidetes (MSP), Firmicutes (MJGT_M), Actinobacteria (MJGT_H) and Proteobacteria (MJGT_H), which predominated the rat intestinal flora system (>99% in total) among treatments (LDA > 4, *p* < 0.05, Figure 6b and Figure 7). In the NC group, Firmicutes and Bacteroides occupied the largest proportions, 85.8% and 9.3%, respectively, which agreed with the major flora of other mammalian intestines [23]. After administration, the percentage of Firmicutes decreased to 75.8% (MSP), 82.1% (MJGT_L) and 77.1% (MJGT_H), respectively, whereas the percentage of the latter phylum increased to 20.5% (MSP), 16.1% (MJGT_L) and 11.0% (MJGT_H). Interestingly, in the MJGT_M group, opposite variations were observed for Firmicutes and Bacteroides. Except in the MJGT_H group, the species abundance of Proteobacteria decreased after administration, especially in the MJGT_L group, where the maximum reduction was identified (from 3.1% in the NC group to 0.5%). Notably, the percentage of Actinobacteria was lower in the NC (1.1%), MSP (0.7%), MJGT_L (0.5%) and MJGT_M (1.2%) groups, but increased to 7% in the MJGT_H group.

At the family level, distinguished enrichments were also observed. Lachnospiraceae, the most abundant family, was suppressed after treatments, except in MJGT_L group (Figure 6c). Ruminococcaceae remained stable after treatments, but in the MJGT_M group, increased from 24.0% to 35.6%. In MJGT_L group, four families, including Muribaculaceae (6.62% to 11.71%, 1.77-fold), Prevotellaceae (2.25% to 4.16%, 1.85-fold), Lactobacillaceae (2.14% to 5.29%, 2.47-fold) and *Erysipelotricheae * (1.31% to 3.86%, 2.95-fold) showed large increases, but the latter two families reduced (2.14% to 1.34%, 0.63-fold) or kept stable (1.31% to 1.43%) in MSP treatment, respectively. Interestingly, Lactobacillus, a well-known genus for promoting effects on gastrointestinal motility, represented the only member of Lactobacillaceae in this study (Figure S6). Among the five treatments, the Muribaculaceae was significantly enriched in the MSP group (LDA > 4, *p* < 0.05, Figure 7), and Erysipelotricheae and Anaeroplasmataceae in the MJGT_L group (LDA > 4, *p* < 0.01). Notably, Staphylococcaceae was suppressed in both MJGT_L and MJGT_M groups, especially in the former treatment, but was almost doubled after MJGT_H treatment (Figure 6c). The highest concentration treatment also resulted in the enrichment of Corynebacteriaceae (LDA > 4, *p* < 0.05) (Figure 7). Similar contradictory results were observed in the MJGT_L and MJGT_M groups for other families, such as Peptostreptococcacea (Figure 6c).

## 4. Discussion

In the present study, we found that flavonoids, including eriodictyol and luteolin and their glycosides (eriodictyol-7-O-glycoside and luteolin-7-O-glucoside), accounted for approximately 17.67% of the dried MJGT hydro extracts. The eriodictyol-7-O-glycoside was the dominant flavonoid (10.19 ± 0.03% in the dried MJGT hydro extracts or 0.637 mg/mL in MJGT_L hydro extracts). The contents of the glycosides, especially the eriodictyol-7-O-glycoside, were consistent with the observation in the extract of the MJC [24]. Several plants such as *Dracocephalum peregrinum* and *Dracocephalum tanguticum* in the *Dracocephalum* genus are also rich in eriodictyol and the corresponding glycosides [25]. The low temperature cultivation is crucial to boost the content of eriodictyol-7-O-glycosides in D. peregrinum that was in agreement with the high-altitude environment where the MJC grows. The eriodictyol-7-O-glycoside is an ideal free radical scavenger [26], and is able to confer protection against cisplatin-induced toxicity by activating the nuclear factor erythroid 2-related factor 2 (Nrf2) [27]. Actions of flavonoids in the digestive system have been reported [28], but no effect of eriodictyol-7-O-glycoside on the gastrointestinal motility was reported.

Flavonoids, especially the eriodictyol-7-O-glycosides in MJGT hydro extracts, would account for the biphasic effect on the gastrointestinal motility in the present study. Three effects of flavonoids on the gastrointestinal motility have been reported. The first is the reduction activity. The quercetin was found to inhibit contraction of guinea pig ileum stimulated by agonists, anaphylaxis or transmural electrical stimulation decades ago [29,30]. The relationship of the inhibitory effect and the molecular structure of flavonoids were also revealed in mice in vivo [31]. For instance, the absence of the 3-hydroxyl group (apigenin, flavone), saturation of the C-2, C-3 double bond (silibinin, taxifolin), saturation of the C-2, C-3 double bond plus removal of the 3-hydroxyl group (naringenin, naringin) and the presence of a 4’-methoxyl group (hesperitin), cyclization of 4,5’-hydroxyl groups (silibinin, silymarin), lack of the C-4 carbonyl (catechin), or opening of the B ring (phloridzin) diminished or abolished the inhibitory effect. In the present study, the saturation of the C-2, C-3 double bond of luteolin diminished the inhibitory effect (eriodictyol). The second effect of flavonoids on gastrointestinal motility regards the promotion effects. Researchers revealed a wide range of luteolin (0.001 to 100 µM) significantly increased the force of the ACh-evoked reaction on abomasum strips of dairy cows, except for the medium concentrations (1 and 10 µM), in which no significant difference was obtained [32]. The best performance occurred at the lowest concentration (0.001 μmol/L). In our luteolin treatments, no enhancements were observed, especially in a low dosage group such as 0.797 μg/mL (S1, 2.784 μmol/L) for any part tested. Conversely, however, the inhibitory effects were observed at medium and higher concentrations [from 1.587 μg/mL (5.545 μmol/L) to 12.030 μg/mL (42.028 μmol/L)]. The third effect is the biphasic effect. Isoliquiritigenin, the crucial component of licorice (*Glycyrrhiza glabra*) root, is both spasmogenic and spasmolytic in the regulation of gastrointestinal motility. At higher concentrations, the prokinetic effect of isoliquiritigenin in the gastric fundus is more predominant than the inhibitory effect on the intestine. Conversely, at low concentrations, the latter is more strongly manifested. The activating of muscarinic receptors would account for the spasmogenic action while the spasmolytic effect is predominantly due to the regulation of α2-adrenergic receptors and blockade of the calcium channels [31,33]. The biphasic effect of quercetin on abomasum strips of dairy cows was also determined [32]. In this study, the biphasic effects were only identified in flavonoid glycosides eriodictyol-7-O-glucoside and luteolin-7-O-glucoside treatments using standard compounds. Compared to the luteolin-7-O-glucoside, the contents of eriodictyol-7-O-glucoside in the MJGT hydro extracts (L, M and H) in vitro tests were more consistent with the concentrations (S3, S4 and S5) of the corresponding standard compounds, respectively. Therefore, the inhibitory effect on gastrointestinal motility during in vitro tests would be attribute to the existence of eriodictyol-7-O-glucoside at high concentrations, whereas the prokinetic effect would result from the contribution of the low or medium concentration of eriodictyol-7-O-glucoside and luteolin-7-*O*-glucoside in MJGT hydro extracts. Lower content of luteolin would also stimulated the gastrointestinal motility, according to the report of Mendel on dairy cows [32], but more evidence is needed to reach such a conclusion regarding rats.

The absorption process of flavonoids is also a major aspect affecting their action and can vary drastically among different flavonoid classes, even for individual compounds in a particular class, which in turn affects bioavailability [34]. Studies have shown that luteolin and its glycosides have higher bioavailability in vivo compared to other flavonoids. For example, the luteolin-7-O-glucoside in ethanolic extract of *Origanum majorana* L. was more bioavailable during the in vitro test of intestinal absorption using a Caco-2 cellular model than luteolin, which may be attributed to their poor permeability capacity into the enterocytes [35]. Higher stability of this glucoside was also observed after absorption. Interestingly, the luteolin can be absorbed more efficiently in the duodenum and jejunum than in the ileum [36]. Our observation supported the conclusion that only the highest concentration of luteolin inhibited the muscle contractility of ileum (12.030 μg/mL) in vitro. It has also been shown that flavonoids containing glucose moieties are significantly more bioavailable than flavonoids with other moieties [37]. Therefore, higher bioavailability in vivo of eriodictyol-7-O-glycosides would also be expected compared to eriodictyol, although more evidence of this is needed.

The activity of MJGT on gastrointestinal motility may also be mediated through the regulation of the expression levels of several key hormones. The specific role of gastrointestinal hormones, such as MTL, GAS and VIP, is to precisely regulate the rate of propulsion of the chyme from the stomach to the duodenum, a process that is essential for further digestion and absorption in the small intestine. The former two hormones are called “accelerating hormones”, whereas the latter (VIP) one is known as the “braking hormone” [38]. Studies have shown that flavonoids regulate the secretion of several gastrointestinal hormones. The flavonoid glycosides in *Polygonum capitatum* reduced the serum levels of IFN-gamma and GAS mouse infected by *Helicobacter pylori* [39]. Intake of proanthocyanidins extracted from grape seed increased the portal levels of active glucagon-like-peptide-1 (GLP-1) and total ghrelin, and decreased the cholecystokinin (CCK) levels that led to the decrease in gastric emptying [40]. In the study, the treatments of MJGT_L and MJGT_M resulted in an increase in MTL and GAS contents with a concomitant decrease for VIP, consistent with gastric emptying and small intestinal propulsion performance in vivo. By contrast, MJGT_H regulated the expression level of MTL, GAS and VIP in an opposite way. The interactions between the components in MJGT and hormones need to be further investigated.

The lower dosage of MJGT hydro extracts favor the proliferation of probiotic bacteria in the gut microbiota. Intake of flavonoids can result in the variation of gut microbiota composition [40,41] – for example, boosting microflora that inhibit the growth of certain pathogens and promote beneficial genera such as Lactobacillus and Bifidobacterium [42]. Recently, the eriodictyol has been shown to protect against intestinal barrier disruption by modulating the balance of intestinal flora [43]. Luteolin mediates the abundance of Lactobacillus, Bacteroides, Roseburia and Butyricicoccus, which are the dominant flora in rats, resulting in a therapeutic effect on gastric ulcers [44]. In this study, low to medium doses of MJGT hydro extracts boosted probiotic bacteria in several families, such as Muribaculaceae, Prevotellaceaeand, Lactobacillaceae and Ruminococcaceae. The population of Muribaculaceae and Ruminococcaceae utilizes the polysaccharides or other carbon sources to produce short-chain fatty acids such as acetate, butyrate and propionate that can be absorbed by host cells and used in the carbohydrate metabolism [45,46,47]. Prevotellaceae members are able to break down dietary fiber, and are especially important for vegetarians [48]. Notably, the important role of gut microbes in the control of intestinal motility was also demonstrated [49,50,51]. For instance, the germ-free rats exhibited a delayed role in gastric emptying compared to normal rats in the presence of enterobacteria [52]. A significant reduction in defecation frequency, as well as a slowing of total intestinal transport time, was also observed [53]. Genus Lactobacillus in Lactobacillaceae affect ghrelin signaling through stabilizing the internalization of ghrelin receptor expressed in the human embryonic kidney cell line [54]. By stimulating the neurotransmitter secretion, *Lactobacillus acidophilus* can improve intestinal transit [55].

The lower dosage of MJGT hydro extracts was able to suppress some pathogenic groups. For instance, Staphylococcaceae is highly aggressive and can colonize the human nasal cavity for long periods. It is also one of the most common causes of skin and soft tissue infections, as well as bloodstream infections, often leading to endocarditis or sepsis [56,57,58]. MJGT_L was found to have a desirable inhibitory effect on Staphylococcaceae. The richness of Proteobacteria, containing a large number of human pathogens such as Brucella, Rickettsia, Bordetella, Neisseria, Escherichia, Shigella and Salmonella, is usually considered a microbial signature of disease; these were also suppressed by the treatment of MJGT_L. Surprisingly, MJGT_H treatment substantially increased the abundance of Staphylococcaceae. The abundant Actinobacteria, the producer of many pharmaceutical compounds, often positively contributes to human health [59]. Interestingly, in this study, the variation of abundance was completely opposite after the administration of MJGT_L and MJGT_H. This phenomenon deserves to be further investigated. Thus, MJGT_L promotes gastrointestinal motility by increasing the abundance of Lactobacillus, while the medium and low concentration groups also promote beneficial bacteria and reduce the production of harmful bacteria.

The Interaction between flavonoids and gut microbiota is complex and mutual. The flavonoids are also extensively metabolized by the gut microbiota [60,61]. Interestingly, *Eubacterium ramulus* in rat gastrointestinal tracts has the ability to transform the luteolin and luteolin-7-O-glucoside to eriodictyol [62], all of which were the dominated compounds in the MJGT hydro extracts. Although the species was not identified in this study, transformations by other microorganisms were inevitable. In fact, approximately 90% of flavonoids after ingestion interact with gut microbiota and the corresponding outcome included types of phenolic intermediates, which subsequently exert multi-functions. Therefore, the specific microbiota enterotypes rather than the concentrations of flavonoids in food play more important roles in the bioavailability [42]. The effect of the structure of different flavonoid molecules on the bioavailability was also not ignored [63]. The complicated relationship among flavonoids, gut microbiota bioactivity and a wide range of metabolites made the investigation on the bioavailability challenging but also fascinating.

## 5. Conclusions

The MJGT hydro extracts were able to regulate the gastrointestinal motility of rats by modulating the contraction of gastrointestinal muscle tissue and regulating the expression level of digestive system-related hormones including MLT, GAS and VIP, and even the gut microbiota. The impact of MJGT is dose-dependent and biphasic. The flavonoid glycosides, eriodictyol-7-O-glucoside and luteolin-7-O-glucoside, dominated the hydro extracts of MJGT and accounted for the biphasic effects on the contraction of gastrointestinal muscle tissue, especially the former glycoside. The dose-response relationship was also reflected in the changes of the gut microbiota of rats. Since only the low and medium doses of MJGT hydro extracts exhibited positive effects on gastrointestinal motility, their usage as a digestive aid could be a noteworthy issue. Similar attentions on other herbal teas should not be ignored.

## Figures and Tables

**Table 1:** Contents of flavonoids in methanol soluble sample of hydro extract of MJGT.

	MJGT/g	Methanol Soluble Sample in Hydro Extract/g	Sample Weight/g	Content Determination			
				Eriodictyol	Eriodictyol-7-O-glucoside	Luteolin	Luteolin-7-O-glucoside
1	8.5365	2.9366	2.956	2.99%	10.23%	0.77%	3.82%
2			3.06	2.94%	10.14%	0.71%	3.72%
3			2.93	2.97%	10.19%	0.74%	3.77%
Mean ± SEM			2.98 ± 0.04	2.97 ± 0.01%	10.19 ± 0.03%	0.74 ± 0.02%	3.77 ± 0.03%

## Data Availability

Data is contained within the article or supplementary material.

## Supplementary Materials

The following supporting information can be downloaded at: https://www.mdpi.com/article/10.3390/foods12040854/s1, Figure S1: UPLC-ESI-MS analytical chromatogram of the MJGT hydro extract at 15.53 (i: eriodictyol-7-*O*-glucoside), 18.10 (ii: luteolin-7-*O*-glucoside), 22.20 (iii: eriodictyol) and 26.84 min (iv: luteolin) of retention time; Figure S2: Structures, suggested fragmentation patterns and full-scanned ion spectra of sofosbuvir. (a), eriodictyol-7-O-glucoside; (b), luteolin-7-O-glucoside; (c), eriodictyol; (d), luteolin; Figure S3: Effects of eriodictyol (a–d), eriodictyol-7-O-glucoside (e–h), luteolin (i–l) and luteolin-7-O-glucoside (m-p) at different concentrations on the frequency of spontaneous activity of the isolated muscle strips from four gastrointestinal tissues in vitro. The frequency of the negative control (NC) was considered as 100%. Capital and lowercase letters above the bar indicate the significant different at 0.01 or 0.05 level (*n* = 10 for each group); Figure S4: Agarose gel electrophoresis analysis of 16S rDNAs; Figure S5: Shannon curve (a) and Sobs index (b) on OUT level; Figure S6: The variations of microbiota composition in genus level; Table S1: The concentrations of hydro extracts or flavonoids used in muscle contractility test in vitro; Table S2: Flavonoids identified by UPLC-ESI-MS in MJGT hydro extracts; Table S3: Regression equations and the corresponding reliability of four flavonoid standards.
