# Supplementary material for: Bidirectional Effects of Mao Jian Green Tea and Its Flavonoid Glycosides on Gastrointestinal Motility
Source: Foods. 2023 Feb 16;12(4):854. doi: 10.3390/foods12040854 (PMC9956896; doi:10.3390/foods12040854)
Supplement: Supplementary file 1 [file foods-12-00854-s001.zip › Table S1.pdf]

Table S1. The concentrations of hydro extracts or flavonoids used in muscle contractility test *in vitro*

Compound (MW)	***Sample***	***Initial concentration (mg/mL)***	***Added volume (μL)***	***Final volume (mL)***	***Final concentration (μg/mL)***	***Final concentration (μmol/L)***
luteolin (286.24)	L	0.034	80.00	20.08	0.135	0.473
	M	0.034	160.00	20.16	0.270	0.943
	H	0.034	320.00	20.32	0.535	1.871
	S1	0.200	80.00	20.08	0.797	2.784
	S2	0.200	160.00	20.16	1.587	5.545
	S3	0.200	320.00	20.32	3.150	11.003
	S4	0.200	640.00	20.64	6.202	21.666
	S5	0.200	1280.00	21.28	12.030	42.028
luteolin-7-O-glucoside (448.38)	L	0.216	80.00	20.08	0.861	1.920
	M	0.216	160.00	20.16	1.714	3.823
	H	0.216	320.00	20.32	3.402	7.587
	S1	0.200	80.00	20.08	0.797	1.778
	S2	0.200	160.00	20.16	1.587	3.539
	S3	0.200	320.00	20.32	3.150	7.025
	S4	0.200	640.00	20.64	6.202	13.832
	S5	0.200	1280.00	21.28	12.030	26.830
eriodictyol (288.25)	L	0.152	80.00	20.08	0.606	2.101
	M	0.152	160.00	20.16	1.206	4.185
	H	0.152	320.00	20.32	2.394	8.304
	S1	0.200	80.00	20.08	0.797	2.764
	S2	0.200	160.00	20.16	1.587	5.507
	S3	0.200	320.00	20.32	3.150	10.927
	S4	0.200	640.00	20.64	6.202	21.514
	S5	0.200	1280.00	21.28	12.030	41.735
eriodictyol-7-O-glucoside (450.39)	L	0.637	80.00	20.08	2.538	5.635
	M	0.637	160.00	20.16	5.056	11.225
	H	0.637	320.00	20.32	10.031	22.273
	S1	0.200	80.00	20.08	0.797	1.769
	S2	0.200	160.00	20.16	1.587	3.524
	S3	0.200	320.00	20.32	3.150	6.993
	S4	0.200	640.00	20.64	6.202	13.769
	S5	0.200	1280.00	21.28	12.030	26.710
