# Supplementary material for: Bidirectional Effects of Mao Jian Green Tea and Its Flavonoid Glycosides on Gastrointestinal Motility
Source: Foods. 2023 Feb 16;12(4):854. doi: 10.3390/foods12040854 (PMC9956896; doi:10.3390/foods12040854)
Supplement: Supplementary file 1 [file foods-12-00854-s001.zip › Table S2.pdf]

Table S2. Flavonoids identified by UPLC-ESI-MS in MJGT hydro extracts

No.	*Peak No.*	*Retetion time*	*Identification Commpond*	*Molecular Weight*	*Ion mode*	*ppm*	*MS*	*MS2*
1	A	15.53	eriodictyol-7-O-glucoside	450.11566	$[M-H]^-$	3.150	449.10828	287.05640/135.04533/151.00388/175.00385
2	B	18.10	luteolin-7-O- glucoside	448.10001	$[M-H]^-$	3.358	447.09369	285.04022/284.03290/327.05228
3	C	22.20	eriodictyol	288.06284	$[M-H]^-$	4.708	287.05627	107.01394/135.04533/151.00385
4	D	26.84	luteolin	286.04719	$[M-H]^-$	4.615	285.04071	133.02963/151.00385/175.04024/199.04041
