# Supplementary material for: Bidirectional Effects of Mao Jian Green Tea and Its Flavonoid Glycosides on Gastrointestinal Motility
Source: Foods. 2023 Feb 16;12(4):854. doi: 10.3390/foods12040854 (PMC9956896; doi:10.3390/foods12040854)
Supplement: Supplementary file 1 [file foods-12-00854-s001.zip › Table S3.pdf]

Table S3. Regression equations and the corresponding reliability of four flavonoid standards

Compound	*Regression equation*	R^2	*Linear ranges (μg/mL)*	*LOD (μg/ml)*	*LQD (μg/ml)*	*Precisio (n=6）*	*Repeatability (n=6）*	*Accuracy(n=6)*	
						RSD (%)	*RSD (%)*	*RSD (%)*	*Recovery (%)*
eriodictyol	Y=32264X-8.1381	0.9998	2.5~250	0.3333	1.0000	1.18%	0.69%	3.22%	94.8%
eriodictyol-7-O-glucoside	Y=23680X+29.012	0.9991	4~400	0.0833	0.3333	1.09%	1.24%	2.24%	96.04%
luteolin	Y=16091X-21.205	0.9995	1.96~196	0.2500	0.8000	1.35%	1.24%	2.13%	98.56%
luteolin-7-O-glucoside	Y=20785X+2.6562	0.9994	2.2~220	0.0270	0.0813	1.70%	1.26%	2.72%	100.58%
