# Supplementary figures and images for: Bidirectional Effects of Mao Jian Green Tea and Its Flavonoid Glycosides on Gastrointestinal Motility
Source: Foods. 2023 Feb 16;12(4):854. doi: 10.3390/foods12040854 (PMC9956896; doi:10.3390/foods12040854)

RT: 0.00 - 30.01

NL:  
8.89E8  
TIC F: FTMS -  
p ESI Full ms  
[100.0000-  
1500.0000]  
MS W1

Relative Abundance

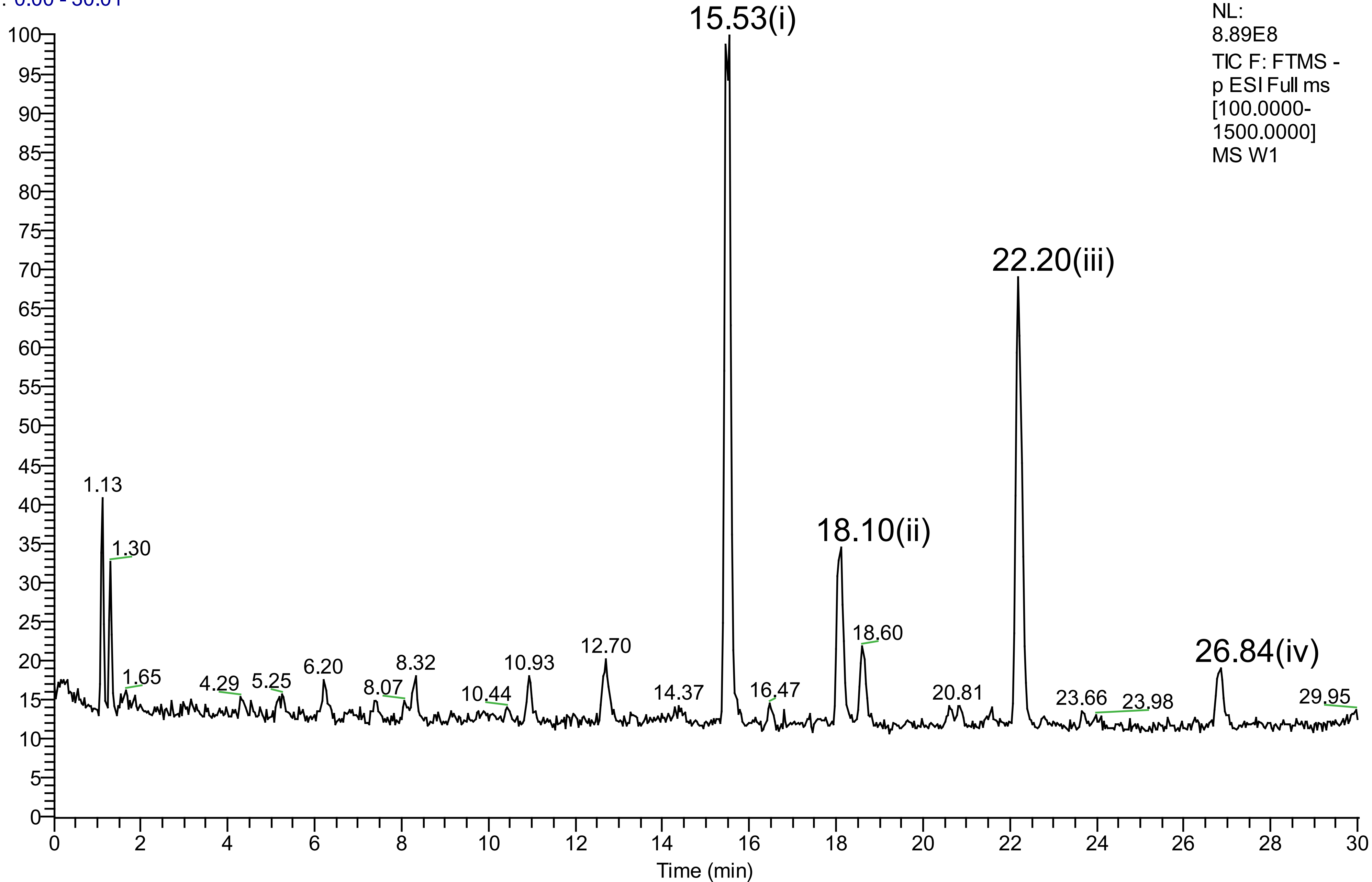

Supplement: Supplementary file 1 [file foods-12-00854-s001.zip › Figure S1.pdf]

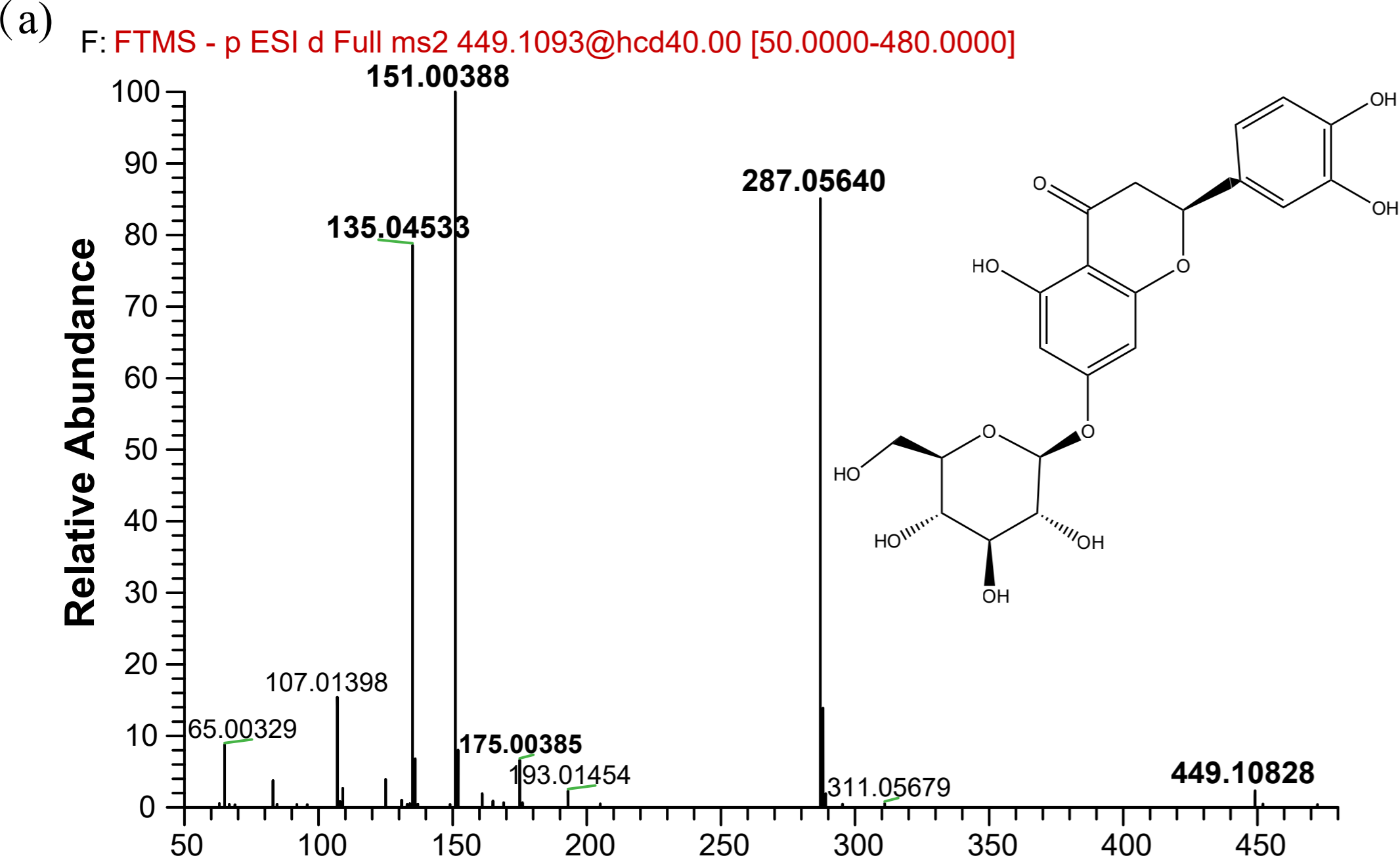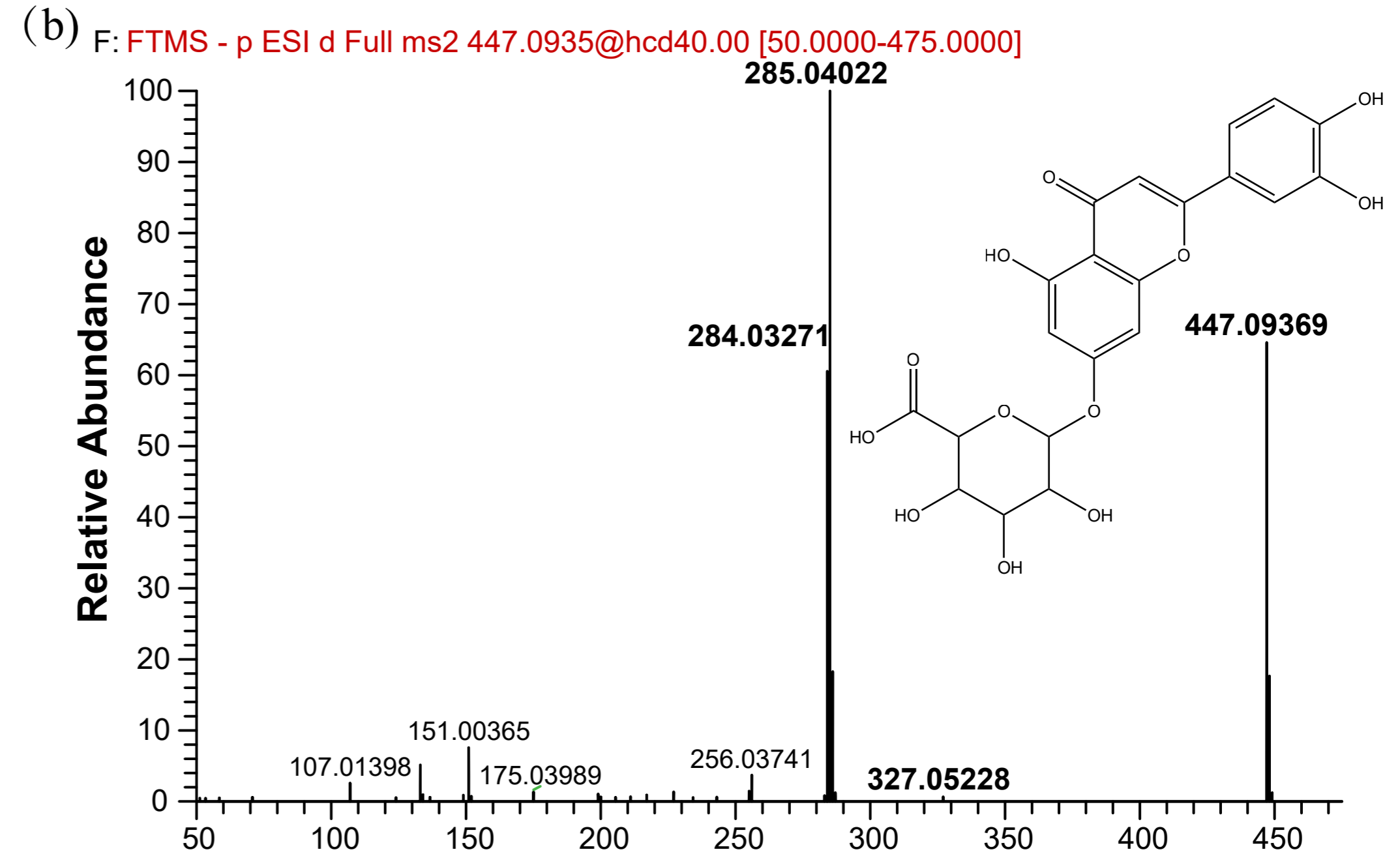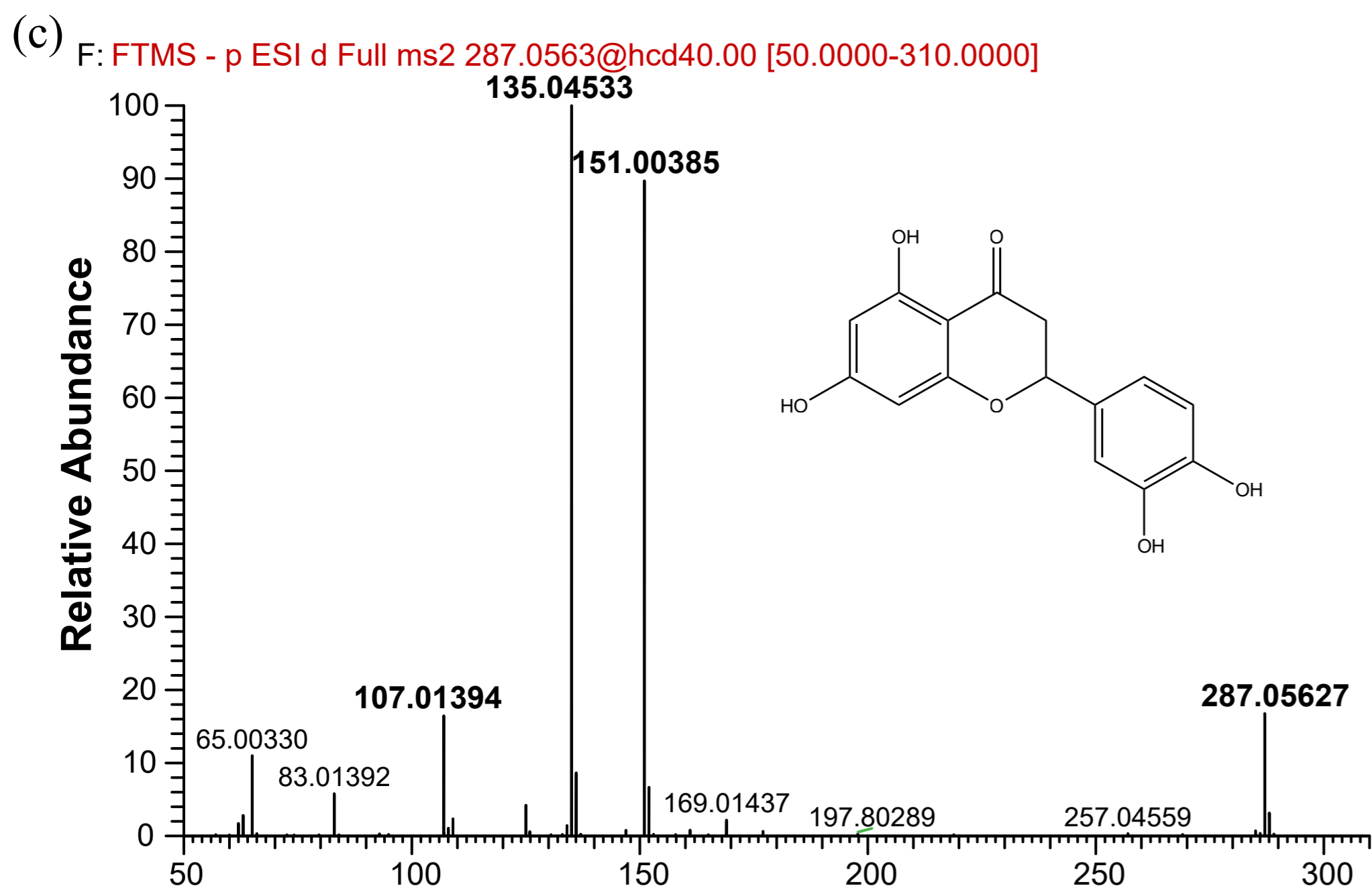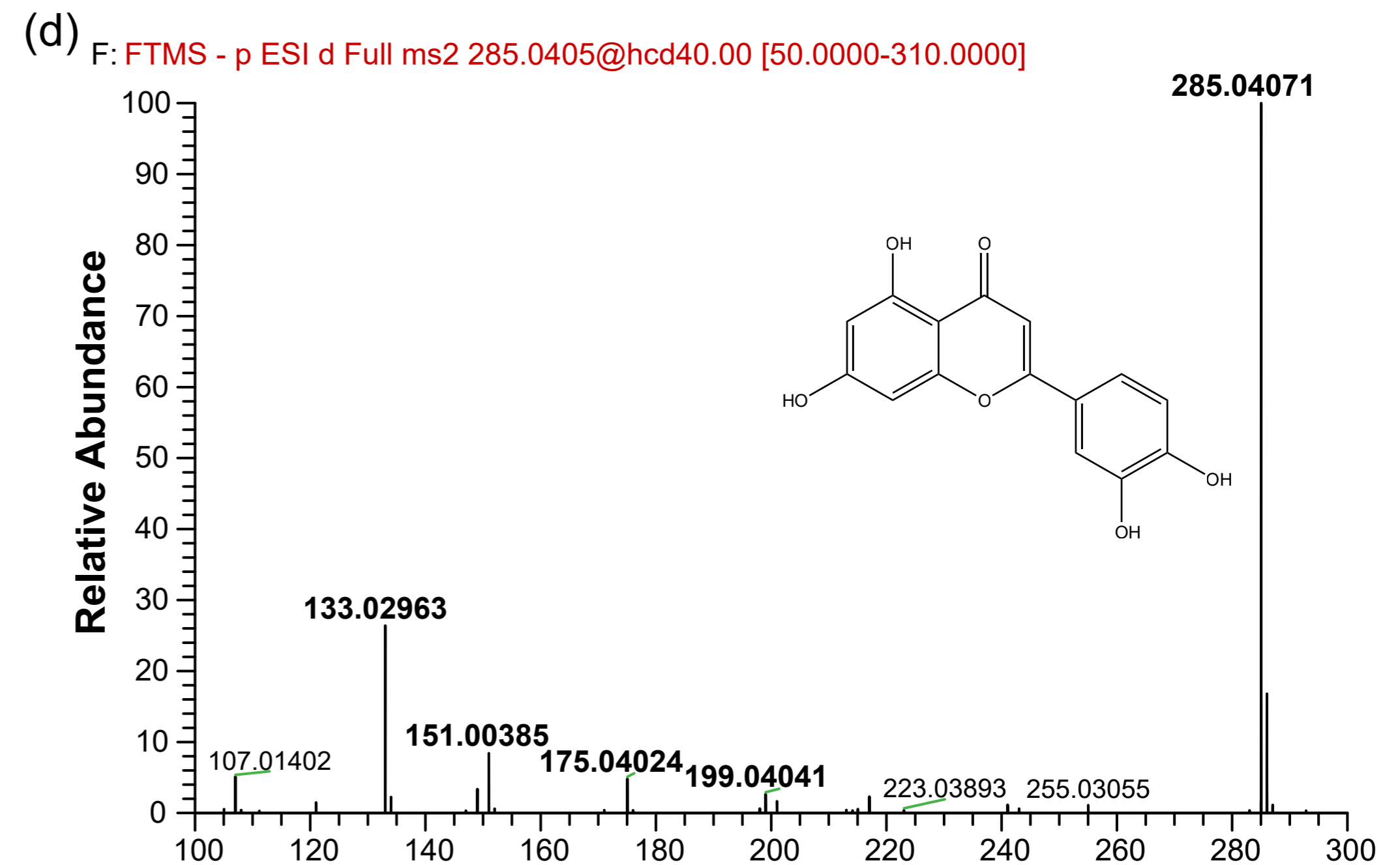

Supplement: Supplementary file 1 [file foods-12-00854-s001.zip › Figure S2.pdf]

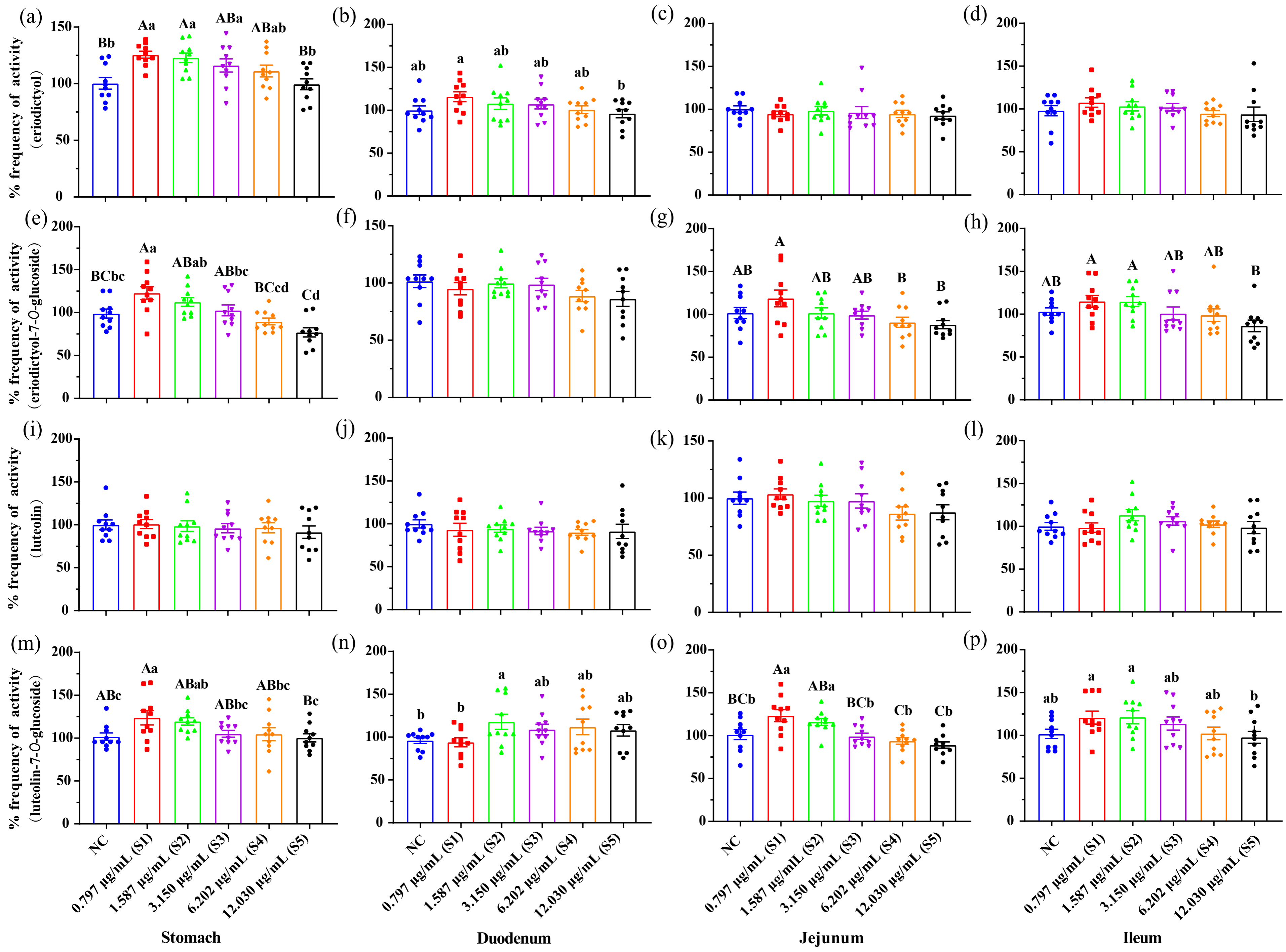

Supplement: Supplementary file 1 [file foods-12-00854-s001.zip › Figure S3.pdf]

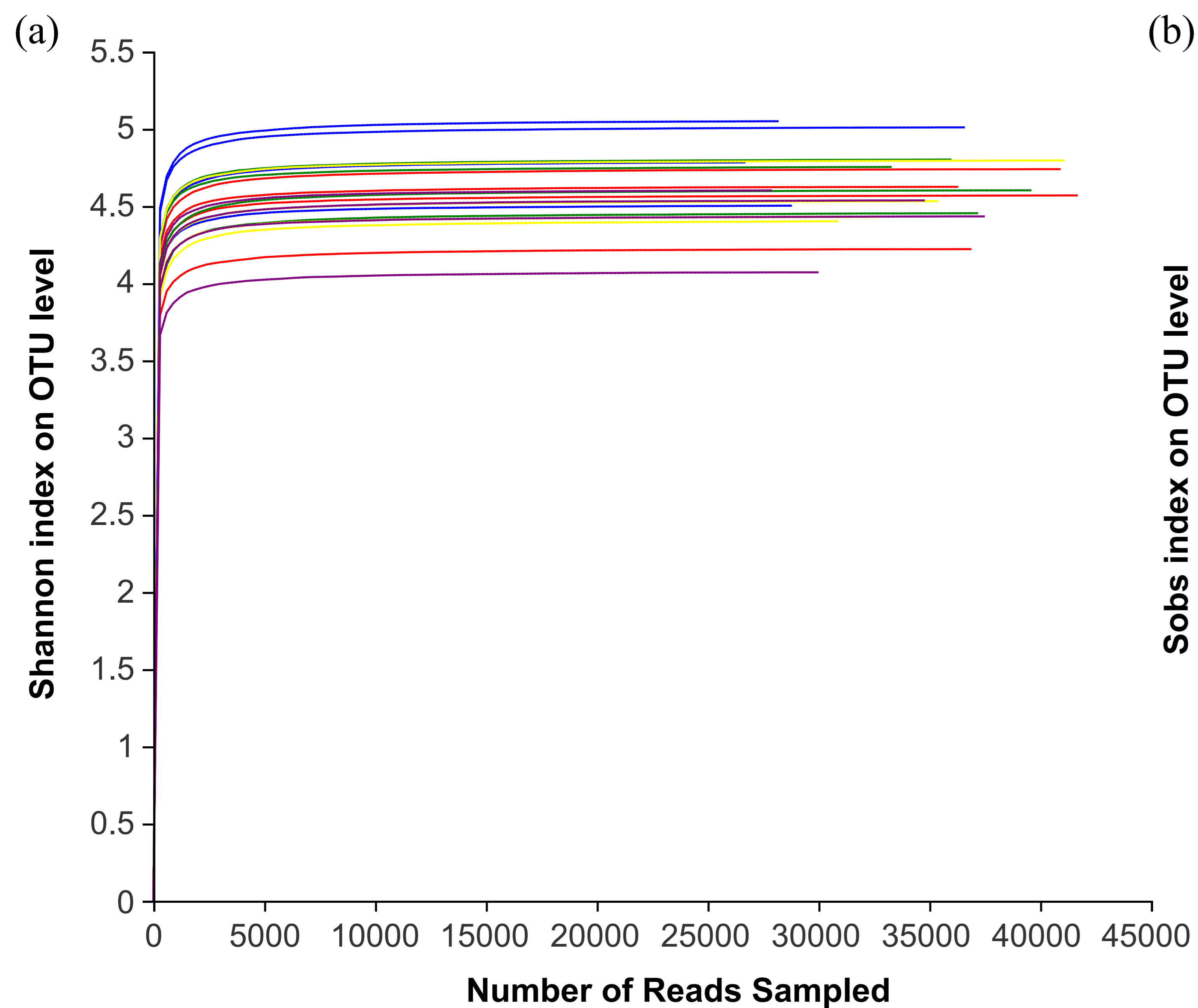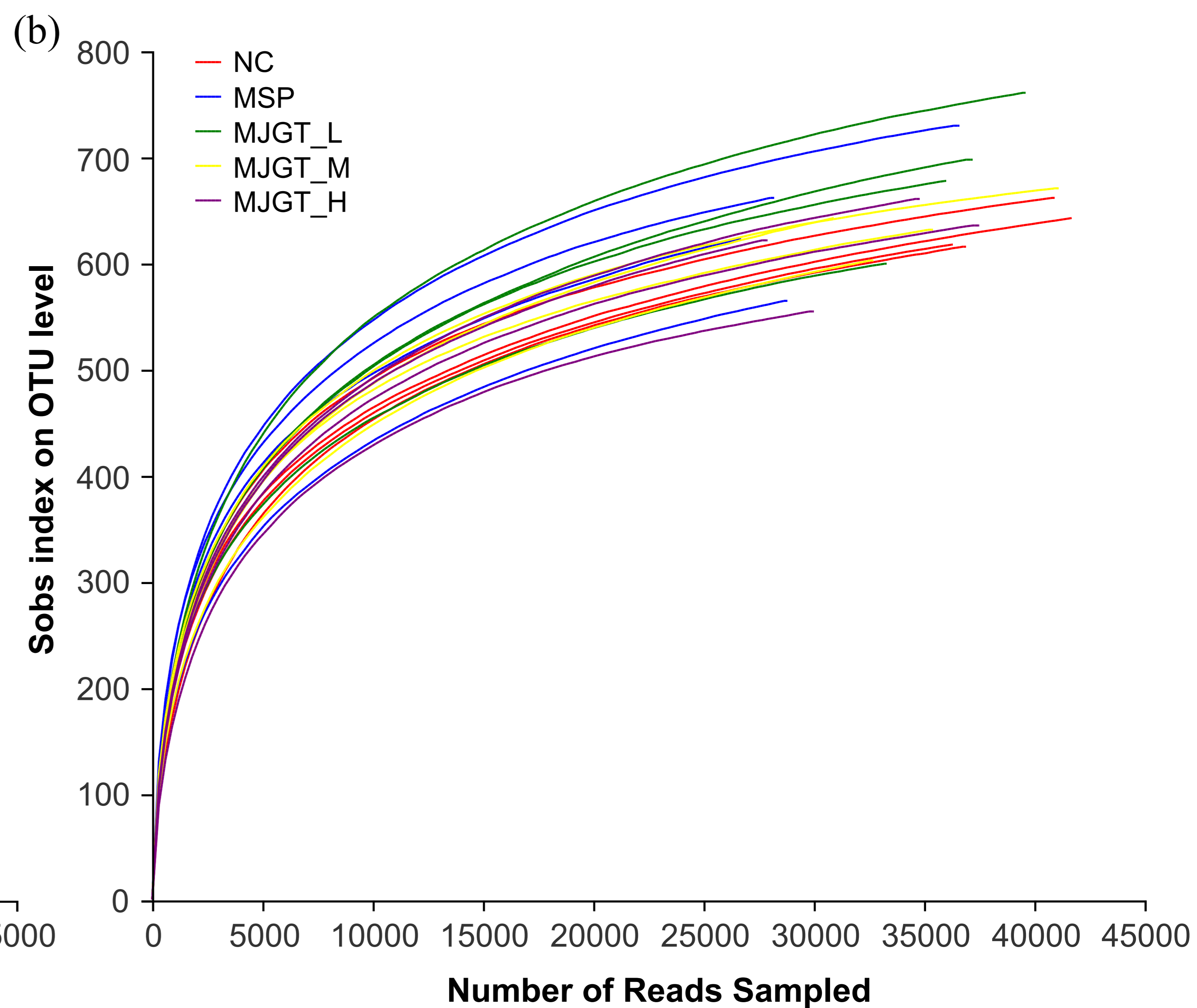

Supplement: Supplementary file 1 [file foods-12-00854-s001.zip › Figure S5.pdf]

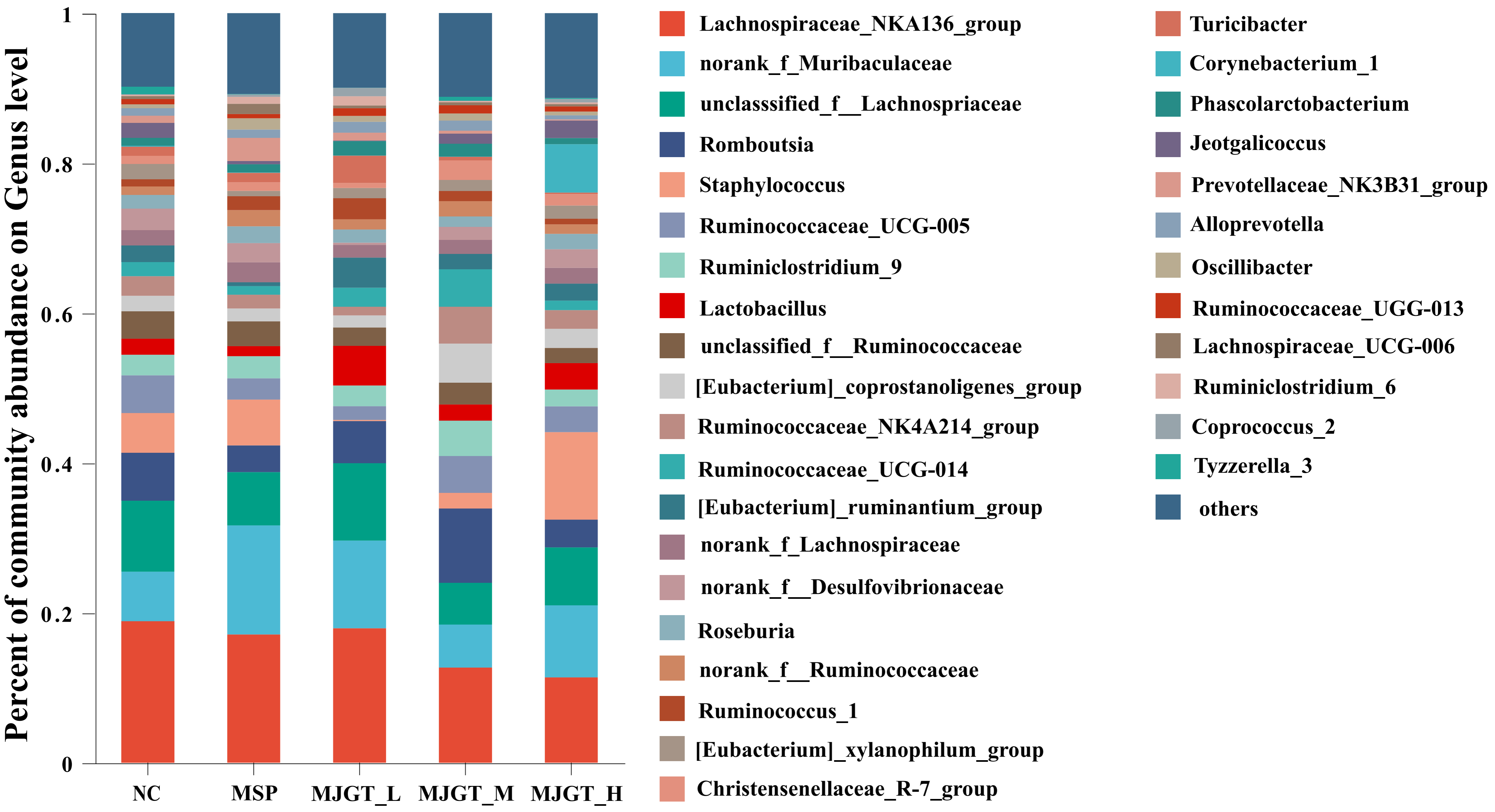

Supplement: Supplementary file 1 [file foods-12-00854-s001.zip › Figure S6.pdf]
